# Subependymal Giant Cell Astrocytoma Non-Associated With Tuberous Sclerosis Complex and Expression of OCT-4 and INI-1: A Case Report

**DOI:** 10.7759/cureus.39187

**Published:** 2023-05-18

**Authors:** Ana-Laura Calderón-Garcidueñas, Steven-Andrés Piña-Ballantyne, Eunice-Jazmín Espinosa-Aguilar

**Affiliations:** 1 Neuropathology, Instituto Nacional de Neurología y Neurocirugía Manuel Velasco Suárez, Mexico City, MEX; 2 Internal Medicine, Clínica-Hospital Mérida, Mérida, MEX

**Keywords:** immunohistochemistry, ini-1, oct-4, tuberous sclerosis complex, subependymal giant cell astrocytoma

## Abstract

Subependymal giant cell astrocytoma (SEGA) is a rare, slow-growing tumor with a dual (neuroglial) component that is typically associated with tuberous sclerosis complex (TSC). We present the case of a healthy 19-year-old man with mild occipital trauma followed by two weeks of intense headache, with no response to analgesics. Imaging studies revealed a well-defined tumor in the left paraventricular zone. A biopsy showed a SEGA (GFAP+, NF+, nestin+, CK-EA3/EA4+, and TTF1+). TSC was ruled out. An immunohistochemistry (IHC) panel showed aberrant cytoplasmic expression of octamer-binding transcription factor 4 (OCT-4) in endothelial cells, pericytes, and some astrocyte-type cells; integrase interactor 1 (INI-1) expression was observed in the cytoplasm of neoplastic cells; SEGA was not associated with TSC; the expression of nestin and OCT-4 suggested their origin in neuroepithelial stem cells; thyroid transcription factor 1 (TTF-1) expression supported its origin in diencephalic structures. Tuberin expression was decreased. An aberrant pattern of INI-1 was observed, which, together with OCT-4 findings, has not been previously described.

## Introduction

The subependymal giant cell astrocytoma (SEGA) is a benign, slow-growing tumor, with a neuroglial component, typically associated with tuberous sclerosis complex (TSC), an autosomal dominant multisystemic hereditary genetic disorder [1]. TSC is associated with mutations in two suppressor genes, TSC1 (9q34.3 gene), which codes for hamartin, and TSC2 (16p113.3), which is for tuberin. Mutations in these genes impact the regulation of cell growth through the mammalian target of rapamycin (mTOR) pathway, inducing cell growth and the development of benign neoplasms [2]. Classified as a circumscribed astrocytic glioma tumor, grade I, according to the WHO in 2021, SEGA is characterized by giant cells of astrocytic type [3].

The largest study of patients with TSC reported that 24.4% developed SEGA. In patients with SEGA, a mutation was detected in 40.6% of them: TSC1 in 10.6% and TSC2 in 89.4% [4].

Although most SEGA cases occur in the context of TSC, there are case reports in which there is no clinical evidence of this pathology. We present a case of a young adult with SEGA that was not associated with TSC, with an immunohistochemistry (IHC) profile that hasn’t been previously described, and may have therapeutic implications.

## Case presentation

The case was approved by the research and ethics committees, and a letter of informed consent was obtained.

A healthy 19-year-old male, college student, suffered an accidental fall and mild trauma to the occipital region, followed by two weeks of intense, pulsatile, and progressive headache that did not respond to analgesics. Owing to this symptom, the patient visited the emergency room.

**Figure 1:**
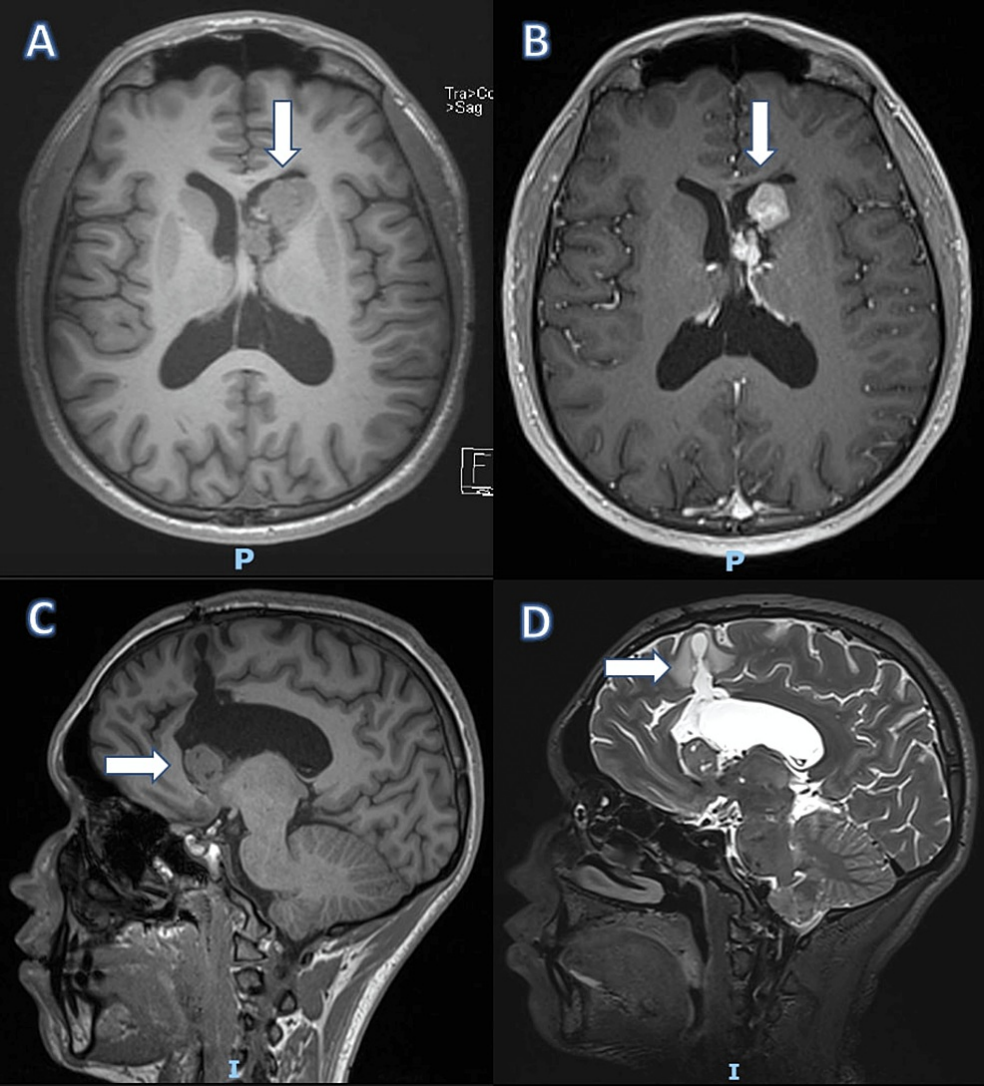
Cranial magnetic resonance imaging in 2019 and 2021 (A) Axial, T1, 2019. (B) Axial, FSPGR (fast spoiled gradient-echo) + gadolinium, 2021. (C) Sagittal, FSPGR, 2019. (D) Sagittal, T2, 2021. (A,C) An intraventricular, isointense, multilobulated tumor (white arrow) at the anterior horn of the left lateral ventricle was observed. (B) With gadolinium, a 3.1 × 1.7 × 2.0 cm tumor exhibited intense and heterogeneous enhancement (white arrow). It was attached to the septum pellucidum and slightly displaced and obliterated the left foramen of Monro. (D) Post-surgical changes were identified (white arrow). When comparing the 2019 and 2021 studies, no substantial changes were observed.

Cranial computed axial tomography (CT scan) showed a well-defined tumor protruding into the left paraventricular zone. The patient was transferred to the surgical room for tumor resection and biopsy at a local hospital. Subsequently, he was referred to our institute for further studies. Family history of TSC was ruled out. General and neurological examinations were normal, including a complete dermatological exam. Original image studies were not available, but magnetic resonance imaging (MRI) showed a residual tumor in the lateral ventricular wall, post-surgical changes, and the absence of cortical tubers or subependymal nodules (Figure 1).

Also, the biopsy was sent for review. In order to complete the analysis, an IHC panel (Table 1) was performed on the material from the paraffin blocks: glial fibrillary acidic protein (GFAP) (MU020-UC, Biogenex, Fremont, CA), neurofilaments (M-0762, Dako, Carpinteria, CA), cytokeratins (CK) AE1/AE3 (PDM072, Diagnostic Biosystems Inc., Pleasanton, CA), Ki67 (MU410-UC, Biogenex, Fremont, CA), signal transducer and activator of transcription 6 (STAT-6) (sc-1689, Santa Cruz Biotechnology, Dallas, TX), nestin (sc-23927, Santa Cruz Biotechnology, Dallas, TX), octamer-binding transcription factor 4 (OCT-4) (NU724-UC, Biogenex, Fremont, CA), hamartin (MC0598, Medaysis, Livermore, CA), tuberin (RC0317, Medaysis, Livermore, CA), integrase interactor 1 (INI-1) (LS-B6039, Life Span BioSciences Inc., Seattle, WA), and thyroid transcription factor 1 (TTF-1) (343M-96, Cell Marque, Rocklin, CA).

**Table 1:** Antibodies used in the immunohistochemistry panel GFAP, glial fibrillary acidic protein; NF, neurofilaments; CK-AE1/AE3, cytokeratins AE1/AE3; STAT-6, signal transducer and activator of transcription 6; OCT-4, octamer-binding transcription factor 4; INI-1, integrase interactor 1; TTF-1, thyroid transcription factor 1

Antibody	Description	Dilution	Company	Catalog number
GFAP	1.0 ml	1:100	Biogenex	MU020-UC
NF	1.0 ml	1:50	Dako	M-0762
CK-AE1/AE3	1.0 ml	1:50	Diagnostic Biosystems Inc.	PDM072
Ki67	1.0 ml	1:10	Biogenex	MU410-UC
STAT-6	1.0 ml	1:50	Santa Cruz Biotechnology	Sc-1689
OCT-4	1.0 ml	1:15	Biogenex	NU724-UC
Nestin	1.0 ml	1:50	Santa Cruz Biotechnology	Sc-23927
Hamartin	1.0 ml	1:50	Medayasis	MC0598
Tuberin	1.0 ml	1:50	Medayasis	RC0317
INI-1	1.0 ml	1:50	Life Span BioSciences Inc.	LS-B6039
TTF-1	1.0 ml	1:100	Cell Marque, Sigma-Aldrich	343-M-96

**Figure 2:**
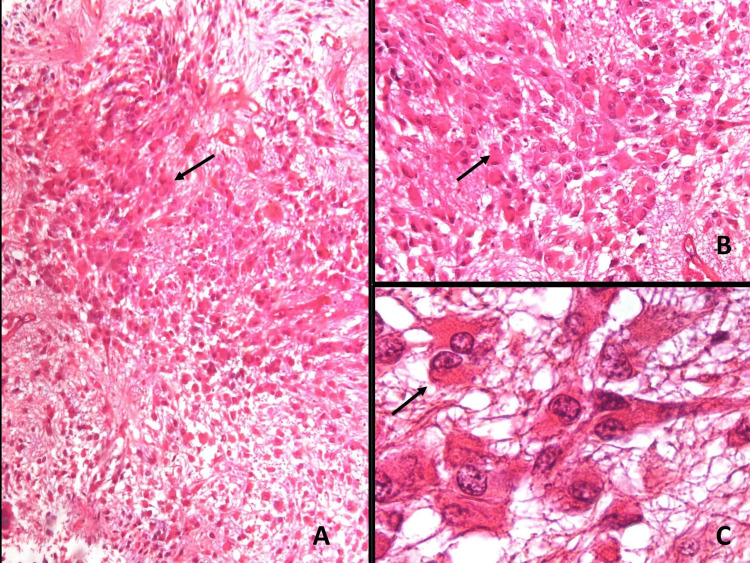
Hematoxylin and eosin stain (100×) Tumor with fibrillar background, large polygonal cells (40-120 m) with abundant eosinophilic cytoplasm, and vesicular nuclei (arrows in A-C) interspersed with elongated fibrillar spindle cells.

Biopsy showed a tumor with an evident fibrillar background, composed of polygonal cells measuring 40-120 microns; with abundant eosinophilic cytoplasm; with fibrillar extensions and vesicular nuclei, frequently eccentric; and with a prominent nucleolus. These cells were interspersed with elongated fibrillar spindle cells with vesicular nuclei that demarcated the nodules from giant cells. The vessels consisted of wide-lumen capillaries and arterioles. In some areas, neoplastic cells adopted a perivascular arrangement. Binucleated cells were observed, but mitosis was not detected (Figure 2).

**Figure 3:**
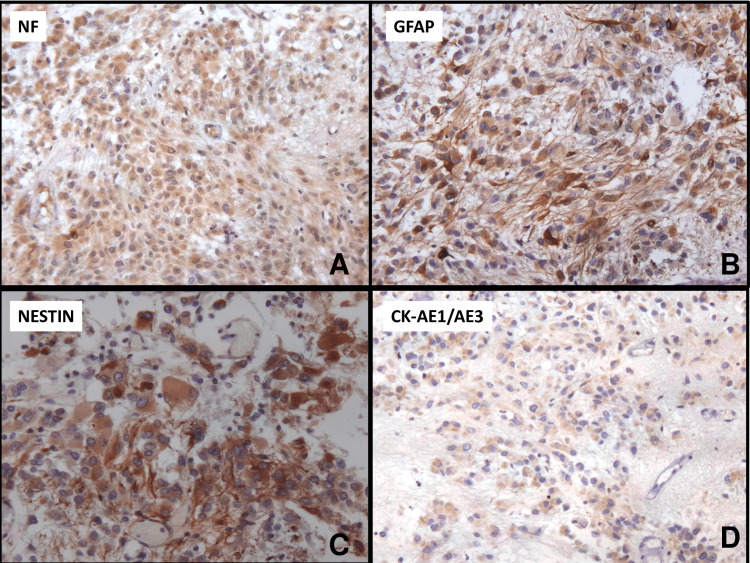
IHC of subependymal giant cell astrocytoma (100×) IHC showed strong cytoplasmic staining with antibodies to NF (A), GFAP (B), nestin (C), and CK (D) NF, neurofilaments; GFAP, glial fibrillary acidic protein; CK, cytokeratins; IHC, immunohistochemistry

Immunohistochemical analysis showed that tumor cells presented a dual profile, with positivity for both neurofilaments (Figure 3A), which showed diffuse positivity in the bodies of the tumor cells, as well as GFAP (Figure 3B), intensely marking the body and cell processes. The cells were also strongly positive for nestin in both the body and the processes (Figure 3C). A positive cytoplasmic stain was detected for CK-AE1/AE3 (Figure 3D).

**Figure 4:**
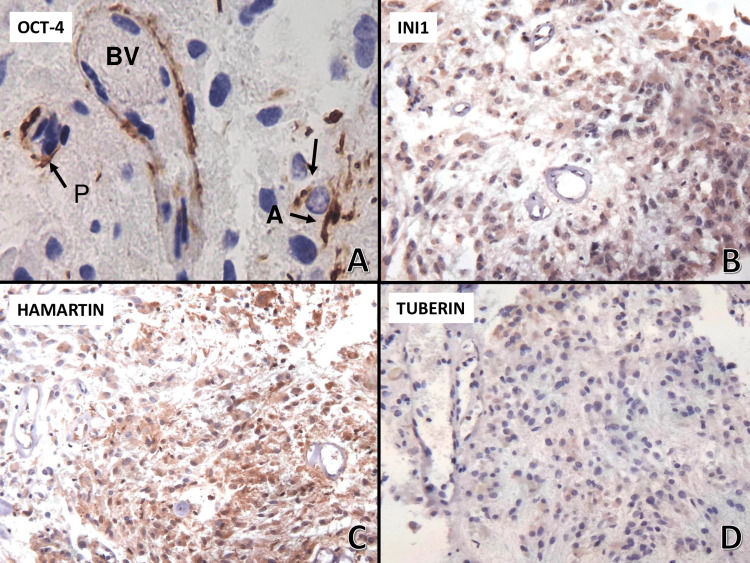
Immunohistochemistry of SEGA (OCT-4 and INI-1) OCT-4 cytoplasmic positivity was observed in endothelial cells, pericytes (P), and astrocytic type-cell (A) appearance, in close contact with giant cell tumor (arrow). Magnification, 400×; tumor cells showed positive cytoplasmic staining with antibodies to INI-1 and hamartin (100×); tuberin showed both positive and negative cytoplasm staining in tumor cells (100×). OCT-4, octamer-binding transcription factor 4; INI-1, integrase interactor 1; SEGA, subependymal giant cell astrocytoma

OCT-4 cytoplasmic positivity was only observed in endothelial cells, subendothelial spindle cells resembling pericytes, and some cells with 20-micron nuclei and radiated branching processes, with an astrocytic appearance, which were observed in close contact with giant cell tumor (Figure 4A). INI-1 did not show nuclear staining but showed diffuse cytoplasmic staining (Figure 4B). Hamartin (Figure 4C) showed diffuse and uniform cytoplasmic staining in all neoplastic cells; however, tuberin showed a mosaic pattern with faint positive cytoplasmic staining and cells with negative staining (Figure 4D). The antibody STAT6 showed intense cytoplasmic positivity in all neoplastic cells, and TTF-1 showed positive nuclear staining (not shown).

A histological review of the tumor concluded that it was a SEGA, and the patient was referred to the Genetics Service to investigate TSC. A full-body CT scan ruled out the presence of cystic and neoplastic lesions in other parts of the body. A next-generation sequencing (NGS) from a blood sample was performed with negative results for TSC1 and TSC2 genes; however, a variant c.-30498A>G, classified as of uncertain significance (VUS), was identified in the APC gene, in a non-canonical promoter region (non-coding site). TSC was ruled out.

Even with the evidence of a tumor on the MRI, the patient remained asymptomatic and neurologically intact. The family rejected a second intervention. Surveillance with neuroimaging studies for every six months was indicated. mTOR inhibitor therapy was not considered for this patient. In the last review, no changes were observed in the MRI between 2019 and 2021. The patient was sent to his hospital of origin for subsequent control and open appointment at our institution, if required.

## Discussion

SEGA accounts for 1-2% of all pediatric brain tumors. SEGA prevalence in patients with TSC is between 6% and 25% [4-6]. In TSC, SEGA is most often diagnosed between 5 and 15 years of age [7]. Although most of these tumors are associated with TSC, there are isolated cases that do not show this association in both children [8] and adults. These cases are usually seen in patients older than 18 years [9] and are usually associated with mutations in the TSC1 and TSC2 genes, at the somatic level [10], or be a “forme fruste” of TSC with somatic mosaic mutations [11]. NGS did not identify mutations in the TSC1 and TSC2 genes but revealed a VUS in the APC gene. Hence, a relationship between the APC gene and SEGA could not be established in this patient; however, a common link could be the mTOR pathway. This pathway is activated in both SEGA and intestinal polyps of a mouse model of familial adenomatous polyposis. In fact, everolimus, an mTOR inhibitor used to decrease tumor volume in patients with SEGA/TSC, showed marked antitumor effects in these mice, targeting both polyp epithelial cells and vascular endothelial cells [12]. On the other hand, we wanted to integrate a panel that supported the neuroepithelial stem cell origin of this tumor, and we used nestin, which in previous articles has been expressed in SEGA [13], and CK-EA1/EA3, which are frequently positive in glioblastomas. The tumor was positive for both proteins, with intense cytoplasmic staining. CK expression had not been previously reported in SEGA, but considering its origin in neuroepithelial stem cells, we decided to explore the possibility of expression, especially in cells with epithelioid characteristics (broad cytoplasm and sharp borders). On the other hand, we wanted to explore transcription factors that participate in the growth control of these tumors. OCT-4 is encoded by the POU5F1 gene in humans. It is a transcription factor that regulates the pluripotency, self-renewal, and maintenance of stem cells. Therefore, it is frequently used as a marker for undifferentiated cells. In this tumor, we observed positive cytoplasmic staining in endothelial cells, cells with morphology and location of pericytes, and cells with radiated processes, astrocyte-type, that touched tumoral cells. OCT-4 is the best-known marker of adult germ cells. In adult mice, ectopic cytoplasmic expression favors the development of cutaneous and intestinal dysplastic lesions, with an increase in the number of progenitor cells, which goes hand in hand with the overexpression of b-catenin and inhibition of cell differentiation [14]. In this tumor, cytoplasmic staining was observed in selected cells. Astrocyte-type cells showed large processes touching the membrane border of giant cells. The cytoplasmic staining of endothelial cells and pericytes may be related to angiogenesis. OCT-4’s participation in SEGA had not been previously described. Perivascular and intratumoral inflammatory infiltrates are commonly observed in SEGA. It is known that Il-4 induces mTOR and STAT6 signaling pathways, activating GATA3 for Th2 cell differentiation. Therefore, we aimed to determine the expression of STAT-6 in this tumor. STAT-6 showed an inactive form with cytoplasmic staining [15].

TTF-1 has recently been described as positive for SEGA. The finding of expression in both SEGA and cells of the developing neuroepithelium in the medial ganglionic eminence suggests that the origin of SEGA is a precursor cell with neoplastic potential in the presence of driving factors [16]. Hamartin is expressed as a normal intense cytoplasmic stain in the tumor tissue. Instead, tuberin presented as a mosaic pattern, with weakly stained cells and others showing negative staining, probably revealing the involvement of the TSC2 gene.

The INI-1 antigen is a product of the INI-1/SMARCB1 gene, which is localized on chromosome 22q. INI-1 showed cytoplasmic positivity in 80% of neoplastic cells, and no nuclear labeling was observed. INI-1 is ubiquitously expressed in the nuclei of normal human cells; however, cytoplasmic staining was observed in this tumor. The INI-1/SMARCB1 gene helps control the growth, division, and death of cells and is considered a tumor suppressor. SEGA is characterized by its cell growth with very large cells grouped in nests and divided by fascicles; it was likely that SEGA had a loss of expression of this marker, and we verified it. However, this finding has not been reported previously.

## Conclusions

In conclusion, the patient had SEGA that was not associated with TSC; the expression of nestin and OCT-4 suggests their origin in neuroepithelial stem cells; TTF-1 expression supports its origin in diencephalic structures. Tuberin expression was decreased. An abnormal pattern of INI-1 was observed, which, together with the OCT-4 findings, had not been previously described. The study of the expression profile of different markers can help to better understand SEGA and therefore facilitate the detection of new therapeutic targets.
